# The 2016 HIV diagnosis and care cascade in New South Wales, Australia: meeting the UNAIDS 90‐90‐90 targets

**DOI:** 10.1002/jia2.25109

**Published:** 2018-04-20

**Authors:** Phillip Keen, Richard T Gray, Barbara Telfer, Rebecca Guy, Heather‐Marie Schmidt, Bill Whittaker, Jo Holden, Martin Holt, Anthony Kelleher, David Wilson, Denton Callander, David A Cooper, Garrett Prestage, Christine Selvey, Andrew E Grulich, the NSW HIV Prevention Partnership Project, Andrew Grulich, Christine Selvey, Rebecca Guy, Garrett Prestage, Iryna Zablotska, Jo Holden, Tim Duck, Craig Cooper, Karen Price, Martin Holt, John de Wit, John Kaldor, Anthony Kelleher, David Wilson, Bill Whittaker, Phillip Keen, Denton Callander, Heather‐Marie Schmidt, Barbara Telfer, Alan Brotherton, Levinia Crooks, David A Cooper

**Affiliations:** ^1^ The Kirby Institute UNSW Sydney Sydney Australia; ^2^ Health Protection NSW Sydney Australia; ^3^ NSW Ministry of Health Sydney Australia; ^4^ Centre for Social Research in Health UNSW Sydney Sydney Australia; ^5^ The Burnet Institute Melbourne Australia

**Keywords:** 90‐90‐90, antiretroviral treatment, continuum of care, HIV, treatment cascade

## Abstract

**Introduction:**

The HIV Strategy in New South Wales (NSW) Australia aims to virtually eliminate HIV transmission by 2020. We estimated the 2016 HIV diagnosis and care cascade for the state of NSW, with a focus on introducing population‐based data to improve data quality and assess progress towards the UNAIDS 90‐90‐90 targets.

**Methods:**

To estimate the number of people living with diagnosed HIV (PLDHIV) we used NSW data from the Australian National HIV Registry, enhanced by surveillance among people recently diagnosed with HIV to improve migration estimates. The number of undiagnosed PLHIV was estimated using back‐projection modelling by CD4 count at diagnosis. De‐duplicated prescription claims data were obtained from the Australian Pharmaceutical Benefits Scheme (PBS), and were combined with an estimate for those ineligible, to determine the number of PLDHIV on antiretroviral therapy (ART). Data from a clinic network with 87% coverage of PLDHIV in NSW enabled the estimation of the number on ART who had HIV suppression.

**Results and discussion:**

We estimated that 10,110 PLHIV resided in NSW in 2016 (range 8400 to 11,720), among whom 9230 (91.3%) were diagnosed, and 8490 (92.0% of those diagnosed) were receiving ART. Among PLDHIV receiving ART, 8020 (94.5%) had suppressed viral load (<200 HIV‐1 RNA copies/mL). Overall, 79.3% of all PLHIV had HIV virological suppression.

**Conclusion:**

NSW has met each of the UNAIDS 90‐90‐90 targets. The enhanced surveillance methods and data collection systems improved data quality. Measuring and meeting the 90‐90‐90 targets is feasible and could be achieved in comparable parts of the world.

## 1 Introduction

In 2014, the Joint United Nations Programme on HIV and AIDS (UNAIDS) launched the 90‐90‐90 targets that by 2020, 90% of people living with HIV (PLHIV) will know their HIV status, 90% of these will receive antiretroviral therapy (ART), and 90% of these will have viral suppression. Overall, this would equate to 73% of all PLHIV being virally suppressed 1.

Several methods have been developed to produce cascade estimates from study populations or by combining population level data 2, 3. A recent systematic review of HIV continua of care (cascade) data reported that only 6 of 53 countries met designated “high quality” criteria, and only Sweden had met all three 90‐90‐90 targets 4. Cascades were classified as high quality if they used all the following: 1 national estimates of PLHIV or estimates from nationally representative surveys and surveillance; 2 a cohort or national programme service database that included everyone diagnosed with HIV; 3 a national cohort or programme service database for those on ART; and 4 individual viral load data for everyone on ART or representative cohort/surveys 4. An earlier systematic review also recommended the use of population‐based data sets to ensure comparability of cascade estimates 5. In 2017, UNAIDS reported that in 2016 Denmark and a small number of so‐called “fast track cities” had also met all three of the 90‐90‐90 targets. In addition, Botswana, Cambodia, Denmark, Iceland, Singapore, Sweden and the United Kingdom had met the overall goal of 73% or more of people living with HIV having viral suppression 6. Also in 2017, results from a cluster‐randomized trial of a universal HIV testing and treatment intervention in East Africa showed that in communities where the intervention was implemented, the UNAIDS population‐level viral suppression target was exceeded 3.

In Australia's most populous state, New South Wales (NSW), the HIV epidemic is concentrated among gay and bisexual men (GBM). Since 1981, 84% of HIV diagnoses have been among men whose reported risk was sex with other men, 3.6% in people who reported injecting drugs and 9.8% among people whose only risk was heterosexual sex 7. In this report, we estimate the HIV cascade in 2016 in NSW and describe enhanced data collection methods.

## 2 Methods

We estimated the three cascade steps, with an estimated upper and lower bound for each, which we termed the plausible range. We provide a summary of the data sources and calculations with full details available from a technical appendix in the HIV, viral hepatitis and sexually transmissible infections in Australia Annual Surveillance Report, 2016 8 and an online repository containing all the code used in the calculations 9.

### 2.1 First 90: the proportion of PLHIV who were diagnosed (PLDHIV)

Australia's HIV surveillance system involves mandatory reporting by doctors and laboratories of all HIV diagnoses, including CD4 count at diagnosis, to state and territory health departments who submit these data to a national HIV registry. Where duplicate cases are reported, cases are allocated to a jurisdiction based on postcode of residence at the earliest reported diagnosis 10. HIV diagnoses among immigrants to Australia are added to the national HIV registry if they have lived in Australia for at least three months and intend to reside in the notifying State/Territory, including among people previously diagnosed overseas 10. Data were provided on NSW cases reported to 31 December 2016. From the notified number of PLDHIV we subtracted duplicates, estimated deaths, and overseas and interstate migration.

All‐cause death rates in PLHIV were based on a linkage study between national HIV and death registers until 2003, and on a large national cohort study of PLHIV since then 11, 12. Two emigration adjustments were applied. First, 6‐month follow up of all people notified with HIV in NSW 13 was used to estimate short‐term emigration. Second, for longer term emigration, and for interstate movement, we used population data from the Australian Bureau of Statistics (ABS) 14. We assumed that long term emigration rates and interstate movement for people living with HIV were the same as for the general population. The plausible range for PLDHIV was estimated by multiplying the appropriate lower and upper bounds for the number of deaths and emigrants. The range in deaths was estimated using the 95% confidence interval of the death rate. We assumed the actual emigration rate ranged between zero and double the estimated long‐term emigration rate in the overall population.

We then applied the European Centre for Disease Prevention and Control (ECDC) HIV Modelling Tool to estimate the proportion of PLHIV who were undiagnosed, and added this to the estimated number of PLDHIV 15. The ECDC tool is a multi‐state back‐calculation model using HIV notification data and estimates for the rate of CD4 decline to fit diagnoses rates over time, which produces an estimate and range of the undiagnosed population and the percentage undiagnosed. The range in PLHIV was obtained by applying the lower estimate for proportion undiagnosed to the lower limit for PLDHIV and conversely for the upper bound.

### 2.2 The second 90: PLDHIV who were receiving ART

We used programme delivery data provided by the Pharmaceutical Benefits Scheme (PBS) on ART prescription claims. The PBS provides a subsidy to cover the cost of ART for people eligible for Medicare in Australia. Only Australian citizens and permanent residents receive this cost subsidy. The PBS collects patient information from pharmacies and produces a de‐duplicated dataset. We used data from NSW residents (based on last registered address) between 1 January and 31 December 2016. The PBS does not subsidize ART for temporary Australian residents, whereas NSW surveillance data includes overseas‐born people diagnosed with HIV if they have lived in Australia for at least three months prior to diagnosis of HIV, and intend to reside in NSW for at least three months 10, 16. For this reason, we added an estimate of the number of HIV‐positive temporary residents in NSW taking ART, based on a survey of clinicians and related cohort study (ATRAS) which estimated there were 450 HIV‐positive temporary residents in Australia in 2014 17. To estimate the number of HIV‐positive temporary residents in NSW taking ART, we first estimated the number of temporary residents living with HIV in NSW by multiplying the national estimate (450) by the cumulative proportion of Australian HIV notifications first diagnosed in NSW (50.2%; giving 226 people). We then assumed 94% of the 180 temporary residents in ATRAS were on treatment (proportion at end of study) and only 63% of the remaining temporary residents were on treatment (matching the baseline proportion on ART from ATRAS). We then calculated the weighted proportion overall (equal to 76%). We then multiplied the estimated population by the proportion on ART. We calculated a range in the number of temporary residents on ART to reflect the uncertainty in the overall population size (400 to 500).

### 2.3 The third 90: PLDHIV on ART who were virally suppressed (<200 copies/mL)

We defined virological suppression as less than 200 viral copies per mL. To estimate the proportion of PLDHIV on ART who were virally suppressed, we used programme service delivery data on last viral load test in 2016 from PLDHIV on ART attending a network of NSW clinics in the Australian Collaboration for Coordinated Enhanced Sentinel Surveillance (ACCESS) project 18. De‐identified patient information from each clinic was collated by ACCESS, and de‐duplicated across clinics. Plausible ranges were estimated by calculating the 95% confidence interval for this proportion. During 2015 to 2016, the ACCESS network was enhanced to enable linkage of patients across clinics, and expanded to include additional GPs, hospitals and sexual health clinics. In 2016, across seven general practice clinics, two hospitals and 33 sexual health clinics, the ACCESS network captured 7998 PLDHIV, 86.7% of all PLDHIV in NSW 19. We estimated the number of PLDHIV on ART with viral suppression by multiplying this proportion by the estimated number of people receiving ART with a range given by the 95% CI of this proportion. One clinic with over 1000 PLDHIV in care was not included in ACCESS but had a similar proportion of patients who were virally suppressed in 2016 as the overall proportion in ACCESS. (Professor Smith, D.E. (online). E‐mail to Phillip Keen (pkeen@kirby.unsw.edu.au) 18 February 2018 (cited 2018 Mar 6)

All calculations were undertaken in R 20. Reproducible code is available online 9 with cleaned input data and raw result files available on request.

## 3 Results

**Figure 1:**
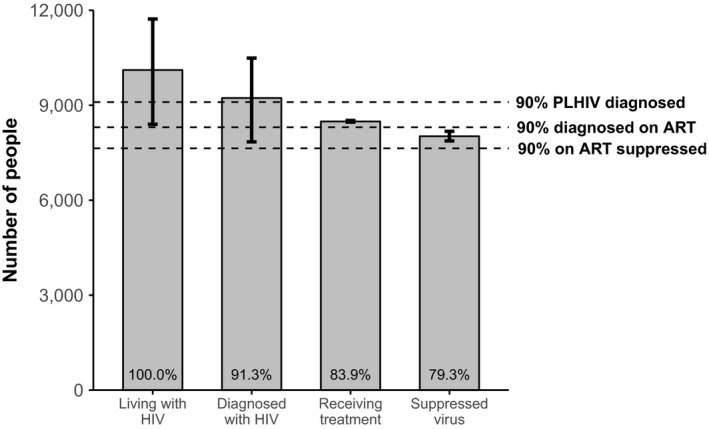
NSW HIV diagnosis and care cascade, 2016, showing the estimated proportion of people living with HIV (PLHIV) at each cascade indicator. Horizontal dotted lines indicate each of the 2014 Joint United Nations Programme on HIV and AIDS (UNAIDS) 90‐90‐90 targets. Columns indicate the number of PLHIV at each cascade step. Numbers within the columns indicate the proportion of all PLHIV at each cascade step. Range bars in columns indicate the estimated plausible range for each cascade step.

Overall, 79.3% (range 67.2 to 97.4%) of all PLHIV in NSW had suppressed virus (Table 1). The NSW 2016 Cascade data are illustrated graphically in Figure 1.

**Table 1:** NSW HIV diagnosis and care cascade estimates to 31 December 2016. Numbers rounded to the nearest 10

Cascade indicator	2016	Percentage of previous cascade indicator (%)^a^	Percentage of all PLHIV (%)
All PLHIV^b^	10,110 (8400 to 11,720)	‐	100
PLDHIV^c^	9230 (8470 to 10,490)	91.3	91.3
Number PLDHIV on ART	8490 (8470 to 8520)	92.0	83.9
Number PLDHIV on ART with suppressed virus^d^	8020 (7870 to 8180)	94.5	79.3

a Percentages of the previous indicator shown correspond to the UNAIDS 90‐90‐90 targets.

b All PLHIV includes the estimated number of undiagnosed and diagnosed people living with HIV.

c PLDHIV is the estimated number of people living with HIV who were diagnosed and residing in NSW.

d The estimated number of people living with HIV who were on ART and had viral suppression (<200 HIV‐1 RNA copies/mL).

### 3.1 First 90: the proportion of PLHIV who were diagnosed

Data from the Australian National HIV Registry showed that 19,099 people had been diagnosed in NSW to 31 December 2016. The ABS 14 estimated that 0.44% of NSW residents emigrated overseas in 2016 17, 21. In follow‐up of people diagnosed with HIV, 50 (4.8%) of 1043 NSW residents newly diagnosed with HIV from 2013 to 2015 had emigrated in the first six months following diagnosis. This short‐term emigration was substantially higher in people born overseas (47 of 486 diagnoses, 9.7%) than in people born in Australia (3 of 557, 0.5%) 13.

Overall, we estimated that by end 2016, 10,110 (range 8400 to 11,720) people were living with HIV in NSW, of whom 9230 (range 8470 to 10,490) or 91.3% were diagnosed. The percentage undiagnosed was 8.7% overall and was lower in men reporting male‐to‐male sex exposure risk (8.4%) than in others (11.5%).

### 3.2 The second 90: PLDHIV who were receiving ART

There were 8315 NSW residents who received subsidized ART through the PBS in 2016. Among Australian temporary residents living with HIV we assumed that the 2014 data were unchanged in 2016 and that they applied to NSW 17, 21 and estimated that there were 172 (range 151 to 201) temporary residents on ART in NSW in 2016 8.

Overall, we estimated that 8490 (range 8470 to 8520) or 92.0% of PLDHIV were receiving ART.

### 3.3 The third 90: PLDHIV on ART who were virally suppressed (<200 copies/mL)

Among 7598 PLDHIV receiving ART in the ACCESS clinic network, representing 89.5% of PLDHIV on ART in NSW, 94.5% were virally suppressed.

Based on this we estimated 8020 (95%CI: 7870 to 8180) of PLDHIV had a suppressed viral load (<200 copies/mL).

## 4 Discussion

In 2016, the UNAIDS 90‐90‐90 targets were exceeded in NSW. Of all PLHIV, 91.3% were diagnosed, 92.0% of those were on ART, and 94.5% of those had suppressed viral load. The percentage of all PLHIV in NSW who had suppressed viral load, of 79.3%, is to the best of our knowledge the highest thus far reported for a jurisdiction in the peer‐reviewed literature. Enhanced systems were developed to utilize population‐level data on ART use, viral load and migration to improve the quality of the NSW cascade.

There have been substantial changes to HIV testing and treatment in NSW in recent years. HIV testing at publicly funded sexual health clinics increased 85% from 30,091 tests in 2014 to 55,761 tests in 2016 7, 22. The percentage of PLDHIV on ART in NSW has also increased rapidly over recent years. In HIV‐positive GBM, self‐reported use of ART increased from 66% in 2006 to 80.6% in 2011 and to 92% in 2016 23, 24, 25. In comparison, the percentage of PLDHIV on ART with suppressed viral load has increased less substantially over time. In 2006, in a large observational cohort of NSW PLDHIV it was already high, at 87% 26.

There are a number of factors which may have contributed to the achievement of the 90‐90‐90 targets in NSW. In NSW, the virtual elimination of HIV by 2020 has been the explicit goal in two consecutive HIV strategies, commencing in 2012 27, 28. Since then, a range of new HIV prevention initiatives aimed at increasing HIV testing and treatment have been implemented. For HIV testing there has been widespread promotion; the introduction of rapid HIV testing at public, private and peer‐led community clinics; and the introduction of streamlined testing services for high‐risk populations. In terms of treatment, Australian clinical guidelines have changed to recommend treatment soon after HIV diagnosis, and there has been a sustained drive to implement these guidelines with aggressive treatment uptake targets in the NSW HIV Strategies 27, 28. There has been education to providers regarding treatment guidelines, and promotion of the benefits of early treatment to PLHIV. Access to HIV treatment has been made easier through dispensing at community pharmacies and co‐payments associated with the receipt of ART have been removed in NSW 27, 28, 29.

In parallel there have been extensive efforts to enhance the quality of data systems. First, the initiation of enhanced surveillance at six months following diagnosis identified that the emigration rate among PLDHIV after diagnosis was higher than previously estimated and we believe this provides a more accurate population estimate of PLHIV 13. These findings are consistent with results of a study in King County, Washington where follow‐up efforts resulted in the identification of PLHIV who had emigrated, resulting in more accurate population estimates 30, 31. Second, rather than relying on patient cohorts or surveys we established systems to provide de‐duplicated programme service delivery data on PBS‐subsidized ART prescription claims, allowing us to report population‐level data on ART. We established a system that extracts de‐identified programme service delivery data on viral load results and de‐duplicates across 42 clinics in a network that was expanded to ensure over 85% coverage of PLDHIV in NSW 7. NSW has been reporting quarterly on the number of people newly diagnosed with HIV who initiate treatment within three and six months of diagnosis 7.

There are a few potential considerations in our data. First, directly collected information on emigration from NSW and Australia were only available for HIV diagnoses from 2013, and only for the first six months after diagnosis and we assumed that longer term migration rates for PLDHIV were the same as general population rates. Second, we assumed that the proportion of PLDHIV who are ineligible for subsidized ART had not changed since 2014, and that the NSW proportion was the same as the Australia‐wide estimate 32. Third, it is possible that viral load suppression rates may be different among a small proportion of PLHIV who are not attending the ACCESS clinics or the additional large clinic we contacted. However, due to the high proportion of PLDHIV included in the ACCESS network and the additional clinic, any lower rates of viral suppression among PLDHIV attending other clinics would have little influence on estimates.

## 5 Conclusions

Based on our estimates, we believe that NSW has met each of the UNAIDS 90‐90‐90 targets and achieved an overall rate of 79.3% of PLHIV with virological suppression. This to the best of our knowledge is the highest rate reported in the peer‐reviewed literature. The enhanced data collection systems introduced in NSW introduced population level data on ART use and viral load that now meet criteria for high‐quality cascade data 4 and could be utilized in other settings. Efforts are continuing to surpass these goals with the current NSW HIV Strategy 2016 to 2020 setting a target to have 95% of all people diagnosed with HIV on ART 28.

## Competing interests

The authors have no conflicts of interest to disclose.

## Authors’ contributions

RTG, RG, PK and AG counceptualized the study and data analysis plan. BT, CS, DC, RG, H‐MS and JH extracted and collected data. RTG, DC, RG and BT performed the data analysis. PK, AG, and RTG drafted the manuscript. RG, H‐MS, BW, AK, MH, DAC, CS and DPW reviewed and commented on initial and final drafts of the manuscript. All authors read and approved the final manuscript.

## Funding

The Kirby Institute and the Centre for Social Research in Health are funded by the Australian Government Department of Health and Ageing. The Kirby Institute is affiliated with the Faculty of Medicine, UNSW, Sydney. This study was funded by a NHMRC Partnership Grant (grant # 1092852), the NSW Ministry of Health, and UNSW, Sydney. The ACCESS study is funded by the Australian Department of Health and Ageing. The content of this publication is solely the responsibility of the authors and does not necessarily represent the official views of any of the institutions mentioned previously.

## Appendix

The investigators on the NSW HIV Prevention Partnership Project are Professor Andrew Grulich, Dr Christine Selvey, Professor Rebecca Guy, Associate Professor Garrett Prestage, Associate Professor Iryna Zablotska, Ms Jo Holden, Mr Tim Duck, Mr Craig Cooper, Ms Karen Price, Professor Martin Holt, Professor John de Wit, Professor John Kaldor, Professor Anthony Kelleher, and Professor David Wilson. The project steering committee includes these investigators and Mr Bill Whittaker, Mr Phillip Keen, Dr Denton Callander, Dr Heather‐Marie Schmidt and Ms Barbara Telfer. The late Mr Alan Brotherton, the late Adjunct Associate Professor Levinia Crooks, and the late Professor David A Cooper were also investigators on the project grant.
